# Supplementary material for: A co-design framework of neural networks and quantum circuits towards quantum advantage
Source: Nat Commun. 2021 Jan 25;12:579. doi: 10.1038/s41467-020-20729-5 (PMC7835384; doi:10.1038/s41467-020-20729-5)
Supplement: Supplementary file 1 — Supplementary Information [file 41467_2020_20729_MOESM1_ESM.pdf]

Supplementary Information to: A Co-Design Framework of Neural Networks and Quantum Circuits Towards Quantum Advantage

Weiwen Jiang[1], Jinjun Xiong[2], and Yiyu Shi[1]

[1]University of Notre Dame, Notre Dame, IN, 46556, USA
[2]IBM Thomas J. Watson Research Center, Yorktown Heights, NY, 10598, USA

Supplementary Note 1 — Details in QuantumFlow

This paper proposes the first co-design framework, namely QuantumFlow, where five sub-components (QF-pNet, QF-hNet, QF-FB, QF-Circ, and QF-Map) work collaboratively to design neural networks and implement them to quantum computers, as shown in Supplementary Figure 1.

In QuantumFlow, the start point is the co-design of shallow networks (i.e., no more than 2 layers) and quantum circuits. We first propose QF-pNet, which contains the probabilistic model based neural computation layer, namely P-LYR. In P-LYR, data are modeled as random variables following a two-point distribution, which is consistent to the expression of a qubit. Thus, computations in P-LYR can be easily implemented by quantum logic gates. Kindly note that P-LYR can model both inputs and weights to be random variables. But because binary weights can achieve comparable high accuracy for deep neural network applications[1] and significantly reduce circuit complexity, we employ random variables for inputs only and binary weights in P-LYR. Benefiting from the quantum-aware data interpretation for inputs, P-LYR can be attached to the output qubits of previous layers without measurement; however, it utilizes 2^k qubits to represent 2^k input data items. Therefore, it has high qubit complexity.

Towards achieving the quantum advantage, we propose a hybrid network, namely QF-hNet, which is composed of two types of neural computation layers: P-LYR and U-LYR. U-LYR is based on the unitary matrix, where $n = 2^k$ inputs are converted to a vector in the unitary matrix, such that all inputs can be represented by the amplitudes of states in a quantum circuit with k qubits. The reduction in input qubits provides the possibility to achieve quantum advantage; however, the state-of-the-art implementation[2] using hypergraph state for computation still has the cost complexity of $O(n \cdot \log n)$. The cost complexity utilized in the paper is the widely used time-space product complexity[3–7]. In this work, we devise a novel optimization algorithm to guarantee the cost complexity of U-LYR to be $O(\log^3 n)$, which makes full use of the properties of neural networks and quantum logic gates. Compared with cost complexity of $O(n)$ on classical computing platforms, U-LYR demonstrates the quantum advantages of executing neural network computations.

In addition to neural computation, batch normalization is commonly employed in neural networks. It contains a normalization step to fix the distribution properties (e.g., means) of layer inputs in a training mini-batch, and thus it can eliminate the

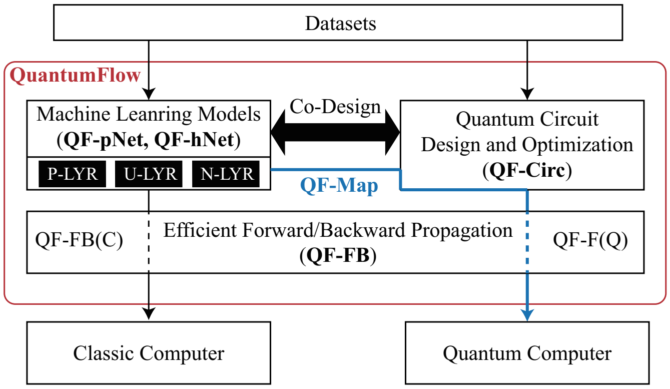

Supplementary Figure 1. QuantumFlow, an end-to-end co-design framework, provides a missing link between neural network and quantum circuit designs, which is composed of QF-pNet, QF-hNet, QF-FB, QF-Circ, QF-Map that work collaboratively to design neural networks and their quantum implementations.

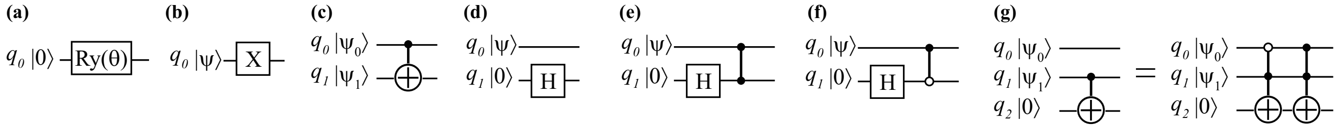

Supplementary Figure 2. Six basic quantum gates and a pair of identity gates in QF-Circ: (a) Ry gate with parameter θ to initialize qubits; (b) Pauli-X (X, or NOT) gate; (c) Controlled X (CNOT) gate to performs the NOT operation on the second qubit only when the first qubit is $|1\rangle$; (d) Hadamard (H) gate acts on qubit q_1 to maps the basis state $|0\rangle$ to $\frac{|0\rangle+|1\rangle}{\sqrt{2}}$; (e) Controlled Z (CZ) gate after H gate; (f) Controlled not Z (CNZ) gate after H gate; (g) identical gates.

internal covariate shift to achieve higher accuracy and accelerate the training procedure. In QF-Nets, we devise a quantum-friendly batch normalization N-LYR, which can be plugged into both QF-pNet and QF-hNet. It includes additional parameters to normalize the output of a neuron, which are tuned during the training phase.

New components in QF-Nets bring new computation paradigm. In order to support both the inference (a.k.a. testing) and training of QF-Nets, we further develop QF-FB, a forward/backward propagation engine. In this work, we apply the classical computer to train the model, denoted as QF-FB(C). The quantum circuits with quantum advantage are designed for accelerating the inference phase, denoted as QF-F(Q). There are two main reasons that we do not consider the training procedure at this stage: first, the training process is a one-time effort while the inference process will be frequently applied; and second, the acceleration of the inference phase will be the base for speeding up the training procedure. The integration of the training procedure in QF-F(Q) will be our future work. In QF-FC, the QF-FB(C) component is integrated into PyTorch for training on classical computers; while QF-F(Q) is implemented based on Qiskit, which can be executed on either quantum processors or Qiskit Aer simulator.

For each operation in QF-Nets (e.g., neural computations and batch normalization), a corresponding quantum circuit is designed in QF-Circ. In neural computation, an encoder is involved to encode the inputs and weights. The output will be sent to the batch normalization which involves additional control qubits to adjust the probability of a given qubit to be ranged from 0 to 1. Based on QF-Nets and QF-Circ, QF-Map is an automatic tool to conduct (1) network-to-circuit mapping (from QF-Nets to QF-Circ); (2) virtual-to-physic mapping (from virtual qubits in QF-Circ to physical qubits in quantum processors). Network-to-circuit mapping guarantees the consistency between QF-Nets and QF-Circ with or without internal measurement; while virtual-to-physic mapping is based on Qiskit with the consideration of error rates.

Finally, we extend the U-LYR to support deep neural networks on a hybrid quantum-classical computing scheme. A quantitative analysis of the cost complexity is conducted to demonstrates the potential quantum advantages to be achieved on both shallow and deep neural networks.

As a whole, given a dataset, QuantumFlow can design and train a quantum-friendly neural network and automatically generate the corresponding quantum circuit. The proposed co-design framework is evaluated on the IBM Qikist Aer simulator and IBM Quantum Processors.

Supplementary Note 2 — Basic Gates used in QF-Circ

In QF-Circ, there are six basic quantum gates, as shown in Supplementary Figure 2(a)-(f). We also utilize a gate identity property in QF-Circ. In this section, we will show the function of each of them.

2.1 Ry gate with parameter θ

Initially, the qubit q_0 in Supplementary Figure 2 is at $|0\rangle$ state, indicating $|q_0\rangle = 1 \cdot |0\rangle + 0 \cdot |1\rangle$. After applying $Ry(\theta)$ on state $|0\rangle$, the state changes to $|q_0\rangle = cos\frac{\theta}{2} \cdot |0\rangle + sin\frac{\theta}{2} \cdot |1\rangle$. q_0 can also be represented as vector: $|q_0\rangle = \begin{bmatrix} \cos\frac{\theta}{2} \\ \sin\frac{\theta}{2} \end{bmatrix}$.

In this case, we have ***i)*** the amplitude of $|0\rangle$ to be $cos\frac{\theta}{2}$; ***ii)*** the amplitude of $|1\rangle$ to be $sin\frac{\theta}{2}$; ***iii)*** the probability of $P\{q_0 = |0\rangle\}$ is $cos^2\frac{\theta}{2}$; and ***iv)*** the probability of $P\{q_0 = |1\rangle\}$ is $sin^2\frac{\theta}{2}$.

2.2 X gate on an initialized qubit

Let q_0 be initialized as $|q_0\rangle = |\psi\rangle = cos\frac{\theta}{2} \cdot |0\rangle + sin\frac{\theta}{2} \cdot |1\rangle$. The X gate is to rotate q_0 around the X-axis by π radians. It is equivalent to conduct operation: $X|\psi\rangle$ where $X = \begin{bmatrix} 0 & 1 \\ 1 & 0 \end{bmatrix}$. After X gate, we have $|q_0\rangle = X|\psi\rangle = \begin{bmatrix} 0 & 1 \\ 1 & 0 \end{bmatrix} \times \begin{bmatrix} \cos\frac{\theta}{2} \\ \sin\frac{\theta}{2} \end{bmatrix} = \begin{bmatrix} \sin\frac{\theta}{2} \\ \cos\frac{\theta}{2} \end{bmatrix}$.

In this case, ***i)*** the amplitudes of $|0\rangle$ and $|1\rangle$ are swapped; ***ii)*** the probability of $P\{q_0 = |0\rangle\}$ and $P\{q_0 = |1\rangle\}$ are swapped.

2.3 CNOT gate on two initialized qubits

Let q_0 be initialized as $|q_0\rangle = |\psi_0\rangle = \begin{bmatrix} \cos\frac{\alpha}{2} \\ \sin\frac{\alpha}{2} \end{bmatrix}$, and q_1 be initialized as $|q_1\rangle = |\psi_1\rangle = \begin{bmatrix} \cos\frac{\beta}{2} \\ \sin\frac{\beta}{2} \end{bmatrix}$. In CNOT operation, q_0 is the control qubit, whose probabilty will not be changed, but q_1 will be rotate around the X-axis by π radians when q_0 is $|1\rangle$.

Before presenting the CNOT operation, we first introduce the combination of states q_0 and q_1 by a tensor product of $|q_0\rangle \otimes |q_1\rangle$, and we have $|q_0, q_1\rangle = |q_0\rangle \otimes |q_1\rangle = \begin{bmatrix} \cos\frac{\alpha}{2} \times \cos\frac{\beta}{2} \\ \cos\frac{\alpha}{2} \times \sin\frac{\beta}{2} \\ \sin\frac{\alpha}{2} \times \cos\frac{\beta}{2} \\ \sin\frac{\alpha}{2} \times \sin\frac{\beta}{2} \end{bmatrix}$ denoted as $\begin{bmatrix} A \\ B \\ C \\ D \end{bmatrix}$, which is $A = \cos\frac{\alpha}{2} \times \cos\frac{\beta}{2}$, $B = \cos\frac{\alpha}{2} \times \sin\frac{\beta}{2}$, $C = \sin\frac{\alpha}{2} \times \cos\frac{\beta}{2}$, and $D = \sin\frac{\alpha}{2} \times \sin\frac{\beta}{2}$. Then we can represent the state of qubits q_0 and q_1 as $|q_0, q_1\rangle = A|00\rangle + B|01\rangle + C|10\rangle + D|11\rangle$. The matrix representation of CNOT is $\begin{bmatrix} 1 & 0 & 0 & 0 \\ 0 & 1 & 0 & 0 \\ 0 & 0 & 0 & 1 \\ 0 & 0 & 1 & 0 \end{bmatrix}$. Therefore, $CNOT|q_0, q_1\rangle = \begin{bmatrix} A \\ B \\ D \\ C \end{bmatrix}$.

In this case, we can see that ***i)*** CNOT swaps the amplitude of $|10\rangle$ and $|11\rangle$. This is accordance with the definition of CNOT, where $|q_1\rangle$ is flipped when $|q_0\rangle = |1\rangle$; therefore, for original state $|11\rangle$ it changes to $|10\rangle$, likewise for original state $|10\rangle$.

2.4 H gate on $|0\rangle$ in a 2-qubits circuit

Let q_0 be initialized as $|\psi_0\rangle = \begin{bmatrix} \cos\frac{\alpha}{2} \\ \sin\frac{\alpha}{2} \end{bmatrix}$. Since $q_1 = |0\rangle = \begin{bmatrix} 1 \\ 0 \end{bmatrix}$, we have $|q_0, q_1\rangle = |q_0\rangle \otimes |q_1\rangle = \begin{bmatrix} \cos\frac{\alpha}{2} \\ 0 \\ \sin\frac{\alpha}{2} \\ 0 \end{bmatrix}$, denoted as $\begin{bmatrix} A \\ 0 \\ C \\ 0 \end{bmatrix}$

Applying H gate on $|q_0, q_1\rangle$ is equal to $I \otimes H|q_0, q_1\rangle$, where $I = \begin{bmatrix} 1 & 0 \\ 0 & 1 \end{bmatrix}$, $H = \frac{1}{\sqrt{2}} \begin{bmatrix} 1 & 1 \\ 1 & -1 \end{bmatrix}$, and $I \otimes H = \frac{1}{\sqrt{2}} \begin{bmatrix} 1 & 1 & 0 & 0 \\ 1 & -1 & 0 & 0 \\ 0 & 0 & 1 & 1 \\ 0 & 0 & 1 & -1 \end{bmatrix}$.

Therefore, after H gate, we have $|q_0, q_1\rangle = \frac{1}{\sqrt{2}} \begin{bmatrix} 1 & 1 & 0 & 0 \\ 1 & -1 & 0 & 0 \\ 0 & 0 & 1 & 1 \\ 0 & 0 & 1 & -1 \end{bmatrix} \times \begin{bmatrix} A \\ 0 \\ C \\ 0 \end{bmatrix} = \frac{1}{\sqrt{2}} \begin{bmatrix} A+0+0+0 \\ A+0+0+0 \\ 0+0+C+0 \\ 0+0+C+0 \end{bmatrix} = \frac{1}{\sqrt{2}} \begin{bmatrix} A \\ A \\ C \\ C \end{bmatrix}$.

In this case, we can see that the function of H gate is to average the amplitude for the pair of $[|00\rangle, |01\rangle]$ and $[|10\rangle, |11\rangle]$.

2.5 CZ gate after H gate

We have already known the state of $|q_0, q_1\rangle$ equals $\frac{1}{\sqrt{2}} \begin{bmatrix} A \\ A \\ C \\ C \end{bmatrix}$. We also have matrix of CZ gate to be $CZ = \begin{bmatrix} 1 & 0 & 0 & 0 \\ 0 & 1 & 0 & 0 \\ 0 & 0 & 1 & 0 \\ 0 & 0 & 0 & -1 \end{bmatrix}$.

After applying CZ gate on $|q_0, q_1\rangle$, we have $CZ|q_0, q_1\rangle = \frac{1}{\sqrt{2}} \begin{bmatrix} 1 & 0 & 0 & 0 \\ 0 & 1 & 0 & 0 \\ 0 & 0 & 1 & 0 \\ 0 & 0 & 0 & -1 \end{bmatrix} \times \begin{bmatrix} A \\ A \\ C \\ C \end{bmatrix} = \frac{1}{\sqrt{2}} \begin{bmatrix} A \\ A \\ C \\ -C \end{bmatrix}$.

In this case, we can see that the function of CZ gate is to make the sign flip on the amplitude of $|11\rangle$.

2.6 CNZ gate after H gate

CNZ gate can be calculated as follows $CNZ = [I \otimes X]CZ[I \otimes X] = \begin{bmatrix} 1 & 0 & 0 & 0 \\ 0 & 1 & 0 & 0 \\ 0 & 0 & -1 & 0 \\ 0 & 0 & 0 & 1 \end{bmatrix}$. Similar with CZ gate, we have

$$CNZ|q_0, q_1\rangle = \frac{1}{\sqrt{2}} \begin{bmatrix} 1 & 0 & 0 & 0 \\ 0 & 1 & 0 & 0 \\ 0 & 0 & -1 & 0 \\ 0 & 0 & 0 & 1 \end{bmatrix} \times \begin{bmatrix} A \\ A \\ C \\ C \end{bmatrix} = \frac{1}{\sqrt{2}} \begin{bmatrix} A \\ A \\ -C \\ C \end{bmatrix}$$

In this case, we can see that the function of CZ gate is to make the sign flip on the amplitude of $|10\rangle$.

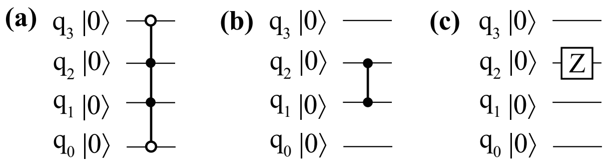

Supplementary Figure 3. Illustration of state $|6\rangle = |0110\rangle$ and $|4\rangle = |0100\rangle$ in a $k = 4$ computation system: (a) FG_6; (b) PG_6; (c) PG_4.

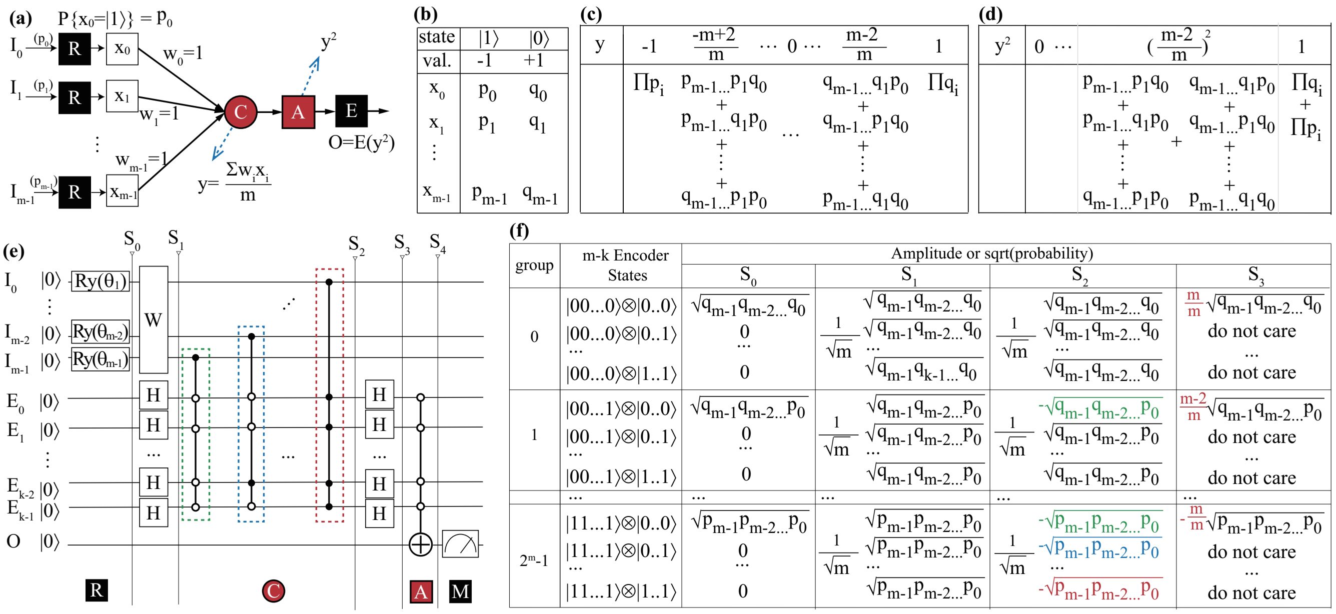

Supplementary Figure 4. Illustration of computation at a neuron in QF-pNet with the square non-linear function: (a) translation to random variables and neural computation; (b)-(d) probability distributions of random variables x, y, and y^2; (e) quantum circuit implementation of neural computation; (f) amplitude of states at different time steps.

2.7 Identical circuits

Two circuits in Supplementary Figure 2(g) are identical. The first gate on the right-hand side circuit having a circle on q_0 and dot on q_1, it indicates that if $q_0 = |0\rangle$ and $q_1 = |1\rangle$, then X gate is operated on q_0. The second Toffoli gate indicate if $q_0 = |1\rangle$ and $q_1 = |1\rangle$, then X gate is operated on q_0. So, what ever $q_0 = |0\rangle$ or $q_0 = |1\rangle$, if $q_1 = |1\rangle$, then X gate is operated on q_2, which is the same function on the left-hand circuit.

2.8 Z gate and Control-Z gates

Finally, the Z gate and Control-Z gates are demonstrate in Supplementary Figure 3.

Supplementary Note 3 — Equivalency of QF-Nets and QF-Circ

Based on the understandings of basic quantum gates, we now discuss the equivalency of QF-pNet and QF-Circ. We will discuss neural computation and batch normalization, respectively.

3.1 Neural Computation

The quantum circuit design of the neural computation in Supplementary Figure 4(a) is illustrated in Figure 4(e). The circuit is composed of m input (I) qubits, and $k = log_2 m$ encoding (E) qubits, and 1 output (O) qubit. The m input qubits and k encoding qubits form a state space with $2^{m+k} = 2^m \times 2^k = m \times 2^m$ states in total. For better understanding, we divide these states into 2^m groups, where each group has the same $|\Phi_0\Phi_1...\Phi_{m-1}\rangle$ state for input qubits and m different states for encoding qubits, as shown in 4(f). We divide the circuit into 5 steps to show the procedure: from S_0 to S_4, as shown in Supplementary Figure 4(e).

At S_0, we initialize the circuit. For input qubit I_k, its initial state Φ_k is obtained by Ry gate by rotating an angle of θ. According to Section 2.1, the probability of $P\{I_k = |1\rangle\} = sin^2\frac{\theta}{2}$, while R operation will generate random variable x_k, where

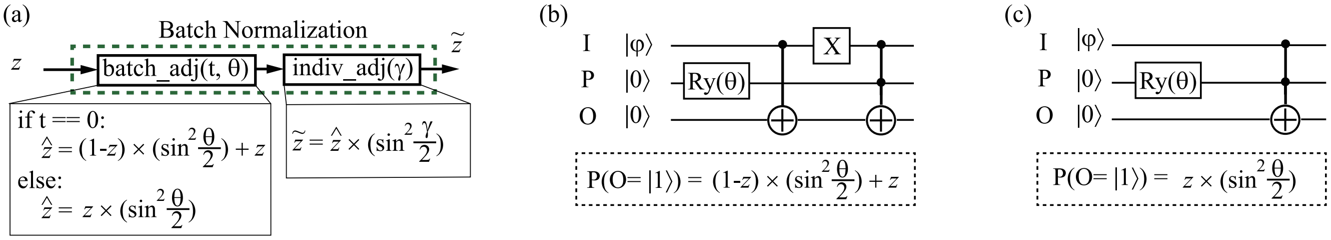

Supplementary Figure 5. Batch normalization in QF-pNet and QF-Circ: (a) functions of "batch_adj" and "indiv_adj"; (b) case $t = 0$ for batch_adj; (c) case $t = 1$ for batch_adj and indiv_ajd if θ is replaced by γ.

the probability that outcomes of -1 (corresponding to sate $|1\rangle$) is the given real number p_k. Hence, we have $sin^2\frac{\theta}{2} = p_k$, and therefore $\theta = 2 \times arcsin(\sqrt{p_k})$. For qubits in encoder from E_0 to E_{k-1}, they are initialized to $|0\rangle$. Hence, at S_0, only the first state in each group has amplitude; i.e., $|\Phi_0\Phi_1...\Phi_{m-1}\rangle \otimes |00...0\rangle$. The amplitude is denoted by A_i for the ith group (e.g., $A_0 = \sqrt{q_{m-1} \cdot q_{m-2} \cdots q_0}$), as shown in Column S_0 in Supplementary Figure 4(f). In addiion, the output qubit is initialized to $|0\rangle$.

From S_0 to S_1, we first conduct dot product of inputs and weight on input qubits. As stated in QF-pNet, if $w_k = -1$, $w_k \cdot x_k$ will leas to the swap of probabilities $P\{x_k = -1\}$ and $P\{x_k = +1\}$ in x_k, which is equivalent to swap $P\{x_k = |1\rangle\}$ and $P\{x_k = |0\rangle\}$. X gate in Section 2.2 has exact the same function, and therefore, a X gate is placed on qubit I_k if and only if $W_k = -1$. For simplify of illustration, we set all weights to 1, and therefore no gates are included in W component. For all encoding qubits, we apply the Hadamard (H) gate to make them enter superposition. From the amplitude perspective, the function of these H gates will averagely distribute the amplitude in each group, as discussed in Section 2.4. The states at S_1 are shown in Column S_1 in Supplementary Figure 4(f).

From S_1 to S_2, the CZ and CNZ gates are performed. As discussed in Sections 2.5 and 2.6, it will lead to the flip of sign for amplitude of specific state. As shown in Supplementary Figure 4(e), each input qubit is associate to one state for the system composed of encoding qubits. Therefore, in the ith group where the input qubits have n_i $|1\rangle$, there are n_i states whose sign is flipped from "+" to "−".

From S_2 to S_3, the circuit completes the creation of all terms in the distribution of y. Here, we only use the information on the first state of each group, i.e., $|\Phi_0\Phi_1...\Phi_{m-1}\rangle \otimes |00...0\rangle$. The function of these H gates on the first state is to accumulate all amplitude in each group and multiplies $\frac{1}{\sqrt{m}}$. As a result, for the ith group where n_i states has flipped sign, the amplitude becomes $\frac{m-2\times n_i}{m} \times A_i$, where A_i is the original amplitude for this state. We denote the amplitude as $B_i \times A_i$ Kindly note that B_i is exactly the same with the corresponding value of random variable y having the probability of A_i^2 in Supplementary Figure 4(c).

Finally, from S_3 to S_4, we apply K-input controlled X gate to extract the information from encoder to output qubit O. According to Section 2.7, the gate adds can be expanded to 2^m gate by traverse all input qubits and encoding qubits, i.e., $|\Phi_0\Phi_1...\Phi_{m-1}\rangle \otimes |00...0\rangle$. For each gate, it indicates that a probability of $(B_i \times A_i)^2$ is added to qubit O. As a result, at S_4, $P\{O = |1\rangle\} = \sum_{\forall i}\{(B_i \times A_i)^2\} = (\frac{m}{m})^2 \cdot q_{m-1}q_{m-2} \cdots q_0 + (\frac{m}{m-2})^2 \cdot q_{m-1}q_{m-2} \cdots p_0 + \cdots + (-\frac{m}{m})^2 \cdot p_{m-1}p_{m-2} \cdots p_0 = E(y^2)$.

Kindly note that the probability of input qubits at step S_4 is exactly the same as that at step S_1. This is because of all H gates applied to the encoding qubits, which will not change the sum of squares of amplitude in a group (i.e., probability). From S_1 to S_2, it only changes the sign of amplitudes. This will also not change the probability since the probability is the sum of the square of amplitudes. In consequence, at state S_2, the probability of all input qubits will not be changed. After S_2, there are no more operations on input qubits, and therefore, the probability of every input qubit at s_4 is the same with that at S_1.

3.2 Batch Normalization

Figure 5 illustrates the proposed batch normalization, whose input is a real number z. We are going to show the equivalency of the functions in Supplementary Figure 5(a) and that in Supplementary Figures 5(b)-(c). First, the parameters of t, θ, γ are determined at training phase.

We first show that the batch_adj circuit for $t = 0$ in Supplementary Figures 5(b) is equivalent to the function in Supplementary Figures 5(a). In the circuit design, we have qubit P for parameter and qubit O for output. The qubit P is initialized using a Ry gate with the determined parameter θ, that is, its probability will be $P\{|P\rangle = |1\rangle\} = sin^2\frac{\theta}{2}$, denoted as P_P. The qubit I is initialized as $P\{|I\rangle = |1\rangle\} = z$, P_I. We first apply a CNOT gate from qubit I to qubit O; then, we apply an X gate on qubit I and then a Toffoli gate using I and P as control. As a result, at the end of the circuit, $|O\rangle = |1\rangle$ if (1) $|I\rangle = |1\rangle$ or (2) $|I\rangle = |0\rangle$ and $|P\rangle = |1\rangle$. The probability of $P\{|O\rangle = |1\rangle\}$ is $P_I + (1 - P_I) \times P_P = z + (1 - z) \times = sin^2\frac{\theta}{2}$, which is the same with that in QF-pNet.

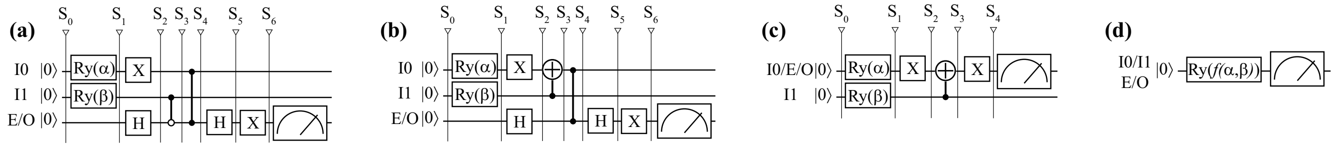

Supplementary Figure 6. Equivalent quantum designs for 2-input neural computation: (a) design 1; (b) design 2; (c) design 3; (d) design 4.

We next show that the circuit in Supplementary Figures 5(c) is equivalent to the function in Supplementary Figures 5(a). Similar to the case of $t = 0$ in batch_adj, the qubit P is initialized to have the probability of $P_P = sin^2\frac{\theta}{2}$. The qubit I is initilized to have probability $P_I = z$. In this circuit, we apply a CNOT gate from qubit I to qubit O; then, we apply a Toffoli gate on qubit O using I and P as control. As a result, at the end of the circuit, $|O\rangle = |1\rangle$ if $|I\rangle = |1\rangle$ and $|P\rangle = |1\rangle$. The probability of $P\{|O\rangle = |1\rangle\}$ is $P_I \times P_P = z \times sin^2\frac{\theta}{2}$, which is the same with that in QF-pNet.

Supplementary Note 4 — Equivalency of Optimized 2-Input Quantum Circuits for Neural Computation

Now, we are ready to demonstrate the equivalency of four quantum circuit designs in Supplementary Figure 6.

4.1 Design 1

In design 1 all qubits are initialized to $|0\rangle$, and then it goes through 6 step to get the final results. In the following, we will show the computing procedure of design 1.

At state $S0$: $|I_0\rangle = \begin{bmatrix}1\\0\end{bmatrix}$; $|I_1\rangle = \begin{bmatrix}1\\0\end{bmatrix}$; $|O\rangle = \begin{bmatrix}1\\0\end{bmatrix}$.

At state $S1$, Ry gate is applied to $|I_0\rangle$ and $|I_1\rangle$: we have $|I_0\rangle = \begin{bmatrix}cos\frac{\alpha}{2}\\ sin\frac{\alpha}{2}\end{bmatrix}$; $|I_1\rangle = \begin{bmatrix}cos\frac{\beta}{2}\\ sin\frac{\beta}{2}\end{bmatrix}$; $|O\rangle = \begin{bmatrix}1\\0\end{bmatrix}$, for the combination of these three qubits, we have $|I_0, I_1, O\rangle = \begin{bmatrix}\cos\frac{\alpha}{2}\times\cos\frac{\beta}{2}\\ 0\\ \cos\frac{\alpha}{2}\times\sin\frac{\beta}{2}\\ 0\\ \sin\frac{\alpha}{2}\times\cos\frac{\beta}{2}\\ 0\\ \sin\frac{\alpha}{2}\times\sin\frac{\beta}{2}\\ 0\end{bmatrix}$, denoted as $\begin{bmatrix}A\\0\\B\\0\\C\\0\\D\\0\end{bmatrix}$.

At state $S2$, we have $|I_0, I_1, O\rangle = X \otimes I \otimes H |I_0, I_1, O\rangle = \frac{1}{\sqrt{2}}\begin{bmatrix}0&0&0&0&1&1&0&0\\0&0&0&0&1&-1&0&0\\0&0&0&0&0&0&1&1\\0&0&0&0&0&0&1&-1\\1&1&0&0&0&0&0&0\\1&-1&0&0&0&0&0&0\\0&0&1&1&0&0&0&0\\0&0&1&-1&0&0&0&0\end{bmatrix} \times \begin{bmatrix}A\\0\\B\\0\\C\\0\\D\\0\end{bmatrix} = \frac{1}{\sqrt{2}}\begin{bmatrix}C\\C\\D\\D\\A\\A\\B\\B\end{bmatrix}$

At state $S3$, the amplitudes of $|X10\rangle$ needs a sign flip, where X can be either 0 or 1; therefore, we have $|I_0, I_1, O\rangle = \frac{1}{\sqrt{2}}\begin{bmatrix}C & C & -D & D & A & A & -B & B\end{bmatrix}^T$

At state $S4$, the amplitudes of $|1X1\rangle$ needs a sign flip; therefore, we have $|I_0, I_1, O\rangle = \frac{1}{\sqrt{2}}\begin{bmatrix}C & C & -D & D & A & -A & -B & -B\end{bmatrix}^T$.

At state $S5$, we apply $I^2 \otimes H = \frac{1}{\sqrt{2}}\begin{bmatrix}1&1&0&0&0&0&0&0\\1&-1&0&0&0&0&0&0\\0&0&1&1&0&0&0&0\\0&0&1&-1&0&0&0&0\\0&0&0&0&1&1&0&0\\0&0&0&0&1&-1&0&0\\0&0&0&0&0&0&1&1\\0&0&0&0&0&0&1&-1\end{bmatrix}$ on $|I_0, I_1, O\rangle$. We have $|I_0, I_1, O\rangle = \begin{bmatrix}C\\0\\0\\-D\\0\\A\\-B\\0\end{bmatrix}$

At state $S6$, we apply $I^2 \otimes X = \begin{bmatrix} 0 & 1 & 0 & 0 & 0 & 0 & 0 & 0 \\ 1 & 0 & 0 & 0 & 0 & 0 & 0 & 0 \\ 0 & 0 & 0 & 1 & 0 & 0 & 0 & 0 \\ 0 & 0 & 1 & 0 & 0 & 0 & 0 & 0 \\ 0 & 0 & 0 & 0 & 0 & 1 & 0 & 0 \\ 0 & 0 & 0 & 0 & 1 & 0 & 0 & 0 \\ 0 & 0 & 0 & 0 & 0 & 0 & 0 & 1 \\ 0 & 0 & 0 & 0 & 0 & 0 & 1 & 0 \end{bmatrix}$, and we have $|I_0, I_1, O\rangle = \begin{bmatrix} 0 \\ C \\ -D \\ 0 \\ A \\ 0 \\ 0 \\ -B \end{bmatrix}$

Finally, we only measure qubit O, which indicate all probability of $|XX1\rangle$ will be sum up. So, the final output probability is $C^2 + (-B)^2 = C^2 + B^2$.

4.2 Design 2

In design 2, only operation between S_2 and S_3 is changed.

We can take use of the previous results at S_2, where $|I_0, I_1, O\rangle = \frac{1}{\sqrt{2}} \begin{bmatrix} C & C & D & D & A & A & B & B \end{bmatrix}^T$.

At $S3$, we apply $CNOT$ on I_0 controller by I_1, indicating all $|X1Y\rangle$ will be swapped to $|\hat{X}1Y\rangle$, where $X = 0$ then $\hat{X} = 1$ or $X = 1$ then $\hat{X} = 0$. Hence, $|I_0, I_1, O\rangle = \frac{1}{\sqrt{2}} \begin{bmatrix} C & C & B & B & A & A & D & D \end{bmatrix}^T$, where B and D swapped.

At $S4$, the amplitudes of $|1X1\rangle$ needs a sign flip; therefore, we have $|I_0, I_1, O\rangle = \frac{1}{\sqrt{2}} \begin{bmatrix} C & C & B & B & A & -A & D & -D \end{bmatrix}^T$.

At $S5$, we will have $|I_0, I_1, O\rangle = \begin{bmatrix} C & 0 & B & 0 & 0 & A & 0 & D \end{bmatrix}^T$ Then, at $S6$, we have $|I_0, I_1, O\rangle = \begin{bmatrix} 0 & C & 0 & B & A & 0 & D & 0 \end{bmatrix}^T$ When we measure qubit O, we can also obtain $C^2 + B^2$.

4.3 Design 3

For design 3, we merge qubit O to $I0$.

At $S1$, we have $|I_0, I_1, O\rangle = \begin{bmatrix} \cos\frac{\alpha}{2} \times \cos\frac{\beta}{2} & \cos\frac{\alpha}{2} \times \sin\frac{\beta}{2} & \sin\frac{\alpha}{2} \times \cos\frac{\beta}{2} & \sin\frac{\alpha}{2} \times \sin\frac{\beta}{2} \end{bmatrix}^T = \begin{bmatrix} A & B & C & D \end{bmatrix}^T$.

Then at $S2$, X gate is applied to $I0$, we swap A, B and C, D to obtain $|I_0, I_1, O\rangle = \begin{bmatrix} C & D & B & A \end{bmatrix}^T$.

Next, at $S3$, CNOT gate is applied to $I0$ controlled by $I1$, hence we swap D and B to obtain $|I_0, I_1, O\rangle = \begin{bmatrix} C & B & A & D \end{bmatrix}^T$.

At $S4$, X gate is applied to $I0$, we swap C, B and A, D to obtain $|I_0, I_1, O\rangle = \begin{bmatrix} A & D & C & B \end{bmatrix}^T$.

Finally, we measure $I0$ which involve the amplitude marked red at $S4$, and we obtain the output probability of $C^2 + B^2$.

4.4 Design 4

For design 4, we take another angle to derive it from design 3.

For design 3, at $S1$ the probability of $P\{I_0 = |1\rangle\}$ is $x = sin^2\frac{\alpha}{2}$, while that of $P\{I_1 = |1\rangle\}$ is $y = sin^2\frac{\beta}{2}$.

At $S2$, the X gate is applied, so the probability of $P\{I_0 = |1\rangle\}$ changes to $1 - x = cos^2\frac{\alpha}{2}$, and $P\{I_0 = |0\rangle\} = x$.

Then at $S3$, if $I_1 = |1\rangle$, I_0 will flip. We know that the probability of $P\{I_1 = |1\rangle\} = y$, so $P\{I_0 = |0\rangle, I_1 = |1\rangle\} = y \times x$ and $P\{I_0 = |1\rangle, I_1 = |1\rangle\} = y \times (1 - x)$. After this step, the probability of $P\{I_0 = |1\rangle\}$ changes to $1 - x + y \times x - y \times (1 - x) = 1 - x - y + 2 \cdot x \cdot y$.

Finally, at $S4$, we flip I_0 again, and the probability of $P\{I_0 = |1\rangle\}$ changes to $1 - [1 - x - y + 2xy] = x + y - 2 \cdot x \cdot y$.

Since x and y are inputs which are known at beginning, we can directly initialize qubit I_0 to obtain the above probability by creating a function $f(\alpha, \beta)$. Let $z = f(\alpha, \beta)$, we know the output probability of design 4 is $sin^2\frac{z}{2}$. We then let $sin^2\frac{z}{2} = x + y - 2 \cdot x \cdot y$, and we can derive $z = 2 \cdot arcsin(\sqrt{x + y - 2 \cdot x \cdot y})$.

Supplementary Note 5 — Demonstration of QF-Nets to QF-Circ

5.1 QF-pNet

QF-pNet in Supplementary Figure 7(a) is a neural network with 2 hidden layers, designed based on two types of layers: neural computation P-LYR and batch normalization N-LYR. In QF-Circ, we notice that the results are stored in state $|1\rangle$ of the output qubits for both P-LYR and N-LYR; while the initialization operation R (using Ry gate) is to encode the previous results to state $|1\rangle$. As a result, P-LYR and N-LYR circuits in QF-Circ can directly take the output qubit from the previous circuit without measurement as input. As such, Supplementary Figure 7(b) shows a corresponding network to QF-Circ, where internal data type conversions in Supplementary Figure 7(a) are removed. Of course, alternatively, BN can still take a given real number (e.g., measured results from the previous circuit) as input, but it would need a Ry gate to initialize qubit I. In this example, we demonstrate the circuits without internal measurement, and we remove the internal data type conversion in Supplementary Figure 7(a) to obtain the network in Supplementary Figure 7(b).

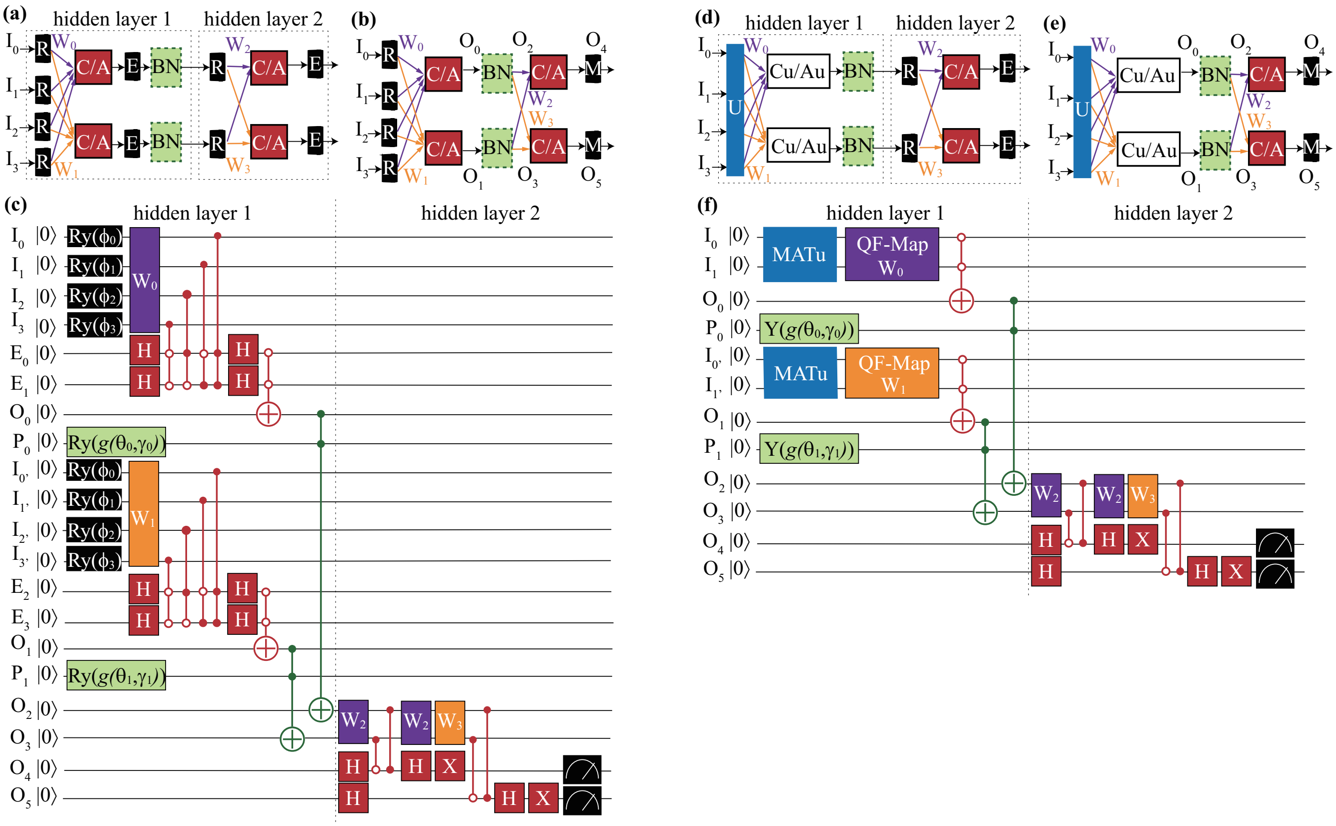

Supplementary Figure 7. Network-to-circuit mapping in QF-Map: (a-c) QF-pNet; (d-f) QF-hNet. (a) QF-pNet with 2 hidden layers for classical computers where batch normalization is applied in the first layer; (b) the corresponding QF-pNet without internal measurement (i.e., no data type conversion); (c) QF-Circ mapped from QF-pNet in (b); (d) QF-hNet with 2 hidden layers for classical computers where batch normalization is applied in the first layer; (e) the corresponding QF-pNet without internal measurement (i.e., no data type conversion); (f) QF-Circ mapped from QF-hNet in (e).

In QF-Circ, operation **R** converts real numbers (inputs) into random variables. To support the efficient simulation on classical computers, we calculate the expectation of the weighted sum of random variables, which requires the inputs are independent. The requirement of independent inputs is not conflicting to that the calculations of neurons in a layer can share the same inputs. However, using the shared inputs at the ith layer will lead the outputs at the ith layer to be dependent. If no measurement can be conducted between layers, we need to duplicate inputs (qubits) for each output neuron in a layer. There is one exception, that is, the last layer. Since the output of the last layer will no longer be used as inputs, there are no requirements of independence for the last layer, and therefore, the last layer can use the shared inputs. As shown in Supplementary Figure 7(c), we utilize two independent sets of qubits ($\{I_0, \cdots, I_3\}$ and $\{I_{0'}, \cdots, I_{3'}\}$) for computing two output neurons using two different weights (W_0 and W_1) in the first layer; while the output qubits of the first layer (i.e., O_2 and O_3) are shared for the calculation of different neurons in the second/last layer.

5.2 QF-hNet

Similar to QF-pNet, we demonstrate the mapping of QF-hNet to QF-Circ in Supplementary Figures 7(d-f). Unlike QF-pNet that uses P-LYR for all layers, QF-hNet employs the U-LYR in the first layer, and P-LYR in the second layer. Supplementary Figures 7(d-e) demonstrate how different neural computations are connected to form a multi-layer network, QF-hNet.

Supplementary Figure 7(f) demonstrates the generated QF-Circ from QF-hNet using QF-Map. Compared to QF-pNet, it is clear that the number of qubits can be reduced from 20 to 12. In addition, in QF-pNet, it required $O(2^k)$ to encode information in input qubits I to the encoder qubits E. On the other hand, the first layer of QF-hNet only need to encode weights to 2 qubits, whose cost can be guaranteed to be $O(k^2)$ by the algorithm in QF-Map.

Benefiting from the reduced size of quantum circuit, we can find a neural network with accuracy of 95.73% for MNIST using less than 16 qubits. For demonstration, we construct it on quirk, and the demos can be accessed via https://wjiang.nd.edu/categories/qf/.

Supplementary Table 1. Complexity of QF-hNet with input size of $O(n)$ on classical and quantum computers.

Network		Perceptron	Shallow Network	
# Neurons in hidden or output layers		O(1)	$O(n^{\frac{1}{k}})$	$O(\log n)$
Classical	Time (T)	$O(1)$	$O(1)$	$O(1)$
	Space (S)	$O(n^2)$	$O(n^{\frac{1}{k}} \cdot n)$	$O(n^{\frac{k+1}{k}})$
	Cost (TS)	$O(n^2)$	$O(n^{\frac{k+1}{k}})$	$O(n\log n)$
**Quantum (QF-hNet)**	Time (T)	$O(\log^2 n)$	$O(n^{\frac{2}{k}} \log n)$	$O(\log^2 n)$
	Space (S)	$O(\log n)$	$O(n^{\frac{1}{k}} \log n)$	$O(\log^3 n)$
	Cost (TS)	$O(\log^3 n)$	$O(n^{\frac{3}{k}} \log^2 n)$	$O(\log^5 n)$
	Quantum Advantage	✓	✓(when $k > 2$)	✓

Supplementary Note 6 — Complexity Analysis of QF-hNet

We first define the cost metric used in the comparison. In general, the time cost and space cost are a pair of tradeoffs. To better describe both costs, we adopt the widely used time-space product complexity, called cost complexity in this paper. In quantum computing, time and space are corresponding to the circuit depth and the number of qubits.

For a fair comparison, in classical computing, time and space are corresponding to computing latency and computing storage. For example, consider a network with d layer, where each layer has $O(n)$ inputs and $O(n)$ outputs. The total storage is $O(d \cdot n^2)$, while in each computation it only needs $O(n^2)$ data. Using computing storage can better describe the tradeoff between time and space. For the above case, if the computing storage complexity is $O(1)$, it requires $O(n^2)$ time for computation, while if the storage complexity is $O(n^2)$, the time complexity can reduce to $O(1)$, indicating parallel execution. In the following discussion, we consider the time complexity to be $O(1)$, that is, all computations in a layer on the classical computing can be conducted in parallel.

The property of QF-hNet is that the whole network is implemented on quantum circuits without measurement. Such a computing paradigm is suitable for the implementation of shallow networks, while for the deep networks, the required qubits will sharply increase along with the increase of layers. This is caused by the requirements of independence among inputs in one layer. Let the number of inputs be $O(n)$, let the number of neurons in hidden layers and output layers be $O(m)$, and let the number of layers be d. Then, space (i.e., qubits) complexity of QF-hNet will be $O(m^{d-1} \times m\log n) = O(m^d \log n)$, where $O(m\log n)$ is the qubits required in the first layer, where inputs are encoded into $\log n$ qubits and m outputs need m sets of inputs to conduct U-LYR. Then, for the latter layers, to guarantee the independence among outputs, the d-layer network needs to be flattened. We have $d-1$ since we relax the independent requirement for the last layer to reduce the number of qubits. Such relaxation will not affect the function correctness since the classification is based on the probability of each output qubit rather than a joint state. Then, according to the time analysis in Table 3 in the manuscript, we know that the time complexity of a basic neural computation is $O(\log^2 n)$ for U-LYR, while it is $O(n \cdot \log n)$ for P-LYR. Considering that P-LYR is applied after the first layer, its complexity is $O(m \cdot \log m)$.

With the space complexity analysis above and the time analysis in Table 3 in the manuscript, we can compare networks with different neurons m and depth d based on QF-hNet between quantum computing and classical computing. Kindly note that, as stated in the manuscript that the quantum state-preparation can be implemented using qRAM, and therefore, the complexity discussed here does not include that complexity.

- $O(m) = O(1)$ and $d = 1$, representing a perceptron. In QF-hNet, it can be implemented using U-LYR only.
  1. On quantum computing, the time complexity of U-LYR is $T = O(\log^2 n)$ and the space complexity is $S = O(\log n)$. Therefore, the cost complexity is $TS = O(\log^3 n)$.
  2. On classical computing, the time complexity is $T = O(1)$ while the space complexity is $S = O(n)$. Therefore, the cost complexity is $TS = O(n)$.
- $O(m) = O(n^{\frac{1}{k}})$ s.t. $k \geq 2$ and $d = 2$, representing a shallow network where the number of neurons in the hidden layer is polynomial times smaller than the inputs.
  1. On quantum computing, we have the time complexity of U-LYR to be $O(\log^2 n)$ and that of P-LYR to be $O(n^{\frac{1}{k}} \log n^{\frac{1}{k}})$; thus, the time complexity is $O(n^{\frac{1}{k}} \log n)$. In addition, its space complexity is $O(n^{\frac{2}{k}} \log n)$ by replacing m to $n^{\frac{1}{k}}$. Therefore, the cost complexity is $O(n^{\frac{3}{k}} \log^2 n)$.
  2. On classical computing, the time complexity is $O(1)$ and the space complexity is $O(n \cdot n^{\frac{1}{k}} + n^{\frac{2}{k}}) = O(n^{\frac{k+1}{k}})$; therefore, the cost complexity is $O(n^{\frac{k+1}{k}})$.
- $O(m) = O(\log n)$ and $d = 2$, representing a shallow network where the number of neurons in the hidden layer is the

logarithm of the inputs.

1. On quantum computing, by replacing m by $\log n$ we have the time complexity is $O(\log^2 n + \log n \log\log n) = O(\log^2 n)$ and the space complexity is $O(\log^3 n)$; therefore, the cost complexity is $O(\log^5 n)$.
2. On classical computing, the time complexity is $O(1)$ and the space complexity is is $O(n\log n + \log^2 n) = O(n\log n)$; therefore, the cost complexity is $O(n\log n)$.

Supplementary Table 1 summarize the complexity for different situations, in terms of neuron numbers m and network depth d. From the table, it is clear to see that for Perceptron (i.e., $d = 1$), QF-hNet can obtain quantum advantage, which is in accordance with the results in Figure 4 in the Manuscript. For the shallow network (i.e., $d = 2$), the quantum advantage can be obtained when $m = n^{\frac{1}{k}}$ and $k > 2$, that is, the neurons in the hidden layer needs to be $O(n^{\frac{1}{k}})$, where n is the input size.

Finally, QF-hNet is designed for shallow neural networks, but it can be extended to support multiple layers. But it has additional costs. Specifically, to guarantee that the outputs of each layer except the last layer have no correlation, we need to flatten the neural network to make sure each output is computed based on an independent input. As a result, the input qubits will exponentially increase along with the network depth; this can be seen from the space complexity $O(m^d \log n)$ at the beginning of this section, where n is the inputs and m neurons in hidden and output layer, and d is the network depth. Kindly note that this is not the limitation of the proposed approach, but generally exists in implementing deep neural networks. A promising and commonly applied solution to this problem is to use the hybrid quantum-classical computing scheme. We have discussed how to apply U-LYR to such a computing scheme in the last subsection of the Method Section.

Supplementary Note 7 — Results Details

Supplementary Table 2. Inference accuracy and efficiency comparison between QF-FB(C) and QF-F(Q) on both QF-pNet and QF-hNet using MNIST dataset which shows the consistency of the inference of QF-Nets on classical computers and quantum computers.

	**QF-pNet**							**QF-hNett**						
	Layer Structure		qubits		Accuracy			Layer Structure		qubits		Accuracy		
dataset	L1	L2	L1	L2	QF-FB(C)	QF-F(Q)	Diff.	L1	L2	L1	L2	QF-FB(C)	QF-F(Q)	Diff.
{3,6}	$16\rightarrow4$	$4\rightarrow2$	28×4	12×2	97.10%	95.53%	-1.57%	$16\rightarrow4$	$4\rightarrow2$	7×4	8×2	98.27%	97.46%	-0.81%
{3,8}	$16\rightarrow4$	$4\rightarrow2$	28×4	12×2	86.84%	83.59%	-3.25%	$16\rightarrow4$	$4\rightarrow2$	7×4	8×2	87.40%	88.06%	+0.54%
{1,3,6}	$16\rightarrow8$	$8\rightarrow3$	28×8	18×3	87.91%	81.99%	-5.92%	$16\rightarrow8$	$4\rightarrow2$	7×8	14×3	88.53%	88.14%	-0.39%

Supplementary Table 3. QuantumFlow demonstrates quantum advantages on neural networks for MNIST datasets with increasing model sizes: comparison on the number of used gates.

Dataset	Structure			MLP(C)			FFNN(Q)				**QF-hNett(Q)**			
	In	L1	L2	L1	L2	Tot.	L1	L2	Tot.	**Red.**	L1	L2	Tot.	**Red.**
{1,5}	16	4	2	132	18	150	80	38	118	**1.27**×	74	38	112	**1.34**×
{3,6}	16	4	2				96	38	134	**1.12**×	58	38	96	**1.56**×
{3,8}	16	4	2				76	34	110	**1.36**×	58	34	92	**1.63**×
{3,9}	16	4	2				98	42	140	**1.07**×	68	42	110	**1.36**×
{0,3,6}	16	8	3	264	51	315	173	175	348	**0.91**×	106	175	281	**1.12**×
{1,3,6}	16	8	3				209	161	370	**0.85**×	139	161	300	**1.05**×
{0,3,6,9}	64	16	4	2064	132	2196	1893	572	2465	**0.89**×	434	572	1006	**2.18**×
{0,1,3,6,9}	64	16	5	2064	165	2229	1809	645	2454	**0.91**×	437	645	1082	**2.06**×
{0,1,2,3,4}	64	16	5				1677	669	2346	**0.95**×	445	669	1114	**2.00**×
{0,1,3,6,9}*	256	8	5	4104	85	4189	5030	251	5281	**0.79**×	135	251	386	**10.85**×

*: Model with 16×16 resolution input for dataset {0,1,3,6,9} to test scalability, whoseaccuracy is 94.09%, which is higher than 8×8 input with accuracy of 92.62%.

References

1. Courbariaux, M., Bengio, Y. & David, J.-P. Binaryconnect: Training deep neural networks with binary weights during propagations. In *Advances in neural information processing systems*, 3123–3131 (2015).

2. Tacchino, F., Macchiavello, C., Gerace, D. & Bajoni, D. An artificial neuron implemented on an actual quantum processor. *npj Quantum Inf.* **5**, 1–8 (2019).

3. Di Matteo, O., Gheorghiu, V. & Mosca, M. Fault-tolerant resource estimation of quantum random-access memories. *IEEE Transactions on Quantum Eng.* **1**, 1–13 (2020).

4. Klauck, H., Špalek, R. & De Wolf, R. Quantum and classical strong direct product theorems and optimal time-space tradeoffs. *SIAM J. on Comput.* **36**, 1472–1493 (2007).

5. Kim, P., Han, D. & Jeong, K. C. Time–space complexity of quantum search algorithms in symmetric cryptanalysis: applying to AES and SHA-2. *Quantum Inf. Process.* **17**, 339 (2018).

6. Frank, M. P. & Ammer, M. J. Relativized separation of reversible and irreversible space-time complexity classes. *arXiv preprint arXiv:1708.08480* (2017).

7. Arute, F. *et al.* Quantum supremacy using a programmable superconducting processor. *Nature* **574**, 505–510 (2019).
